# Cost-effectiveness of programs to eliminate disparities in elderly vaccination rates in the United States

**DOI:** 10.1186/1471-2458-14-718

**Published:** 2014-07-15

**Authors:** Constantinos I Michaelidis, Richard K Zimmerman, Mary Patricia Nowalk, Kenneth J Smith

**Affiliations:** 1 University of Pittsburgh School of Medicine, M240 Scaife Hall, 3550 Terrace Street, Pittsburgh, PA 15261, USA; 2 Department of Family Medicine and Clinical Epidemiology, University of Pittsburgh School of Medicine, Pittsburgh, PA, USA; 3 Section of Decision Sciences and Clinical Systems Modeling, University of Pittsburgh School of Medicine, Pittsburgh, PA, USA

**Keywords:** Vaccination, Elderly, Disparities, Cost-effectiveness

## Abstract

**Background:**

There are disparities in influenza and pneumococcal vaccination rates among elderly minority groups and little guidance as to which intervention or combination of interventions to eliminate these disparities is likely to be most cost-effective. Here, we evaluate the cost-effectiveness of four hypothetical vaccination programs designed to eliminate disparities in elderly vaccination rates and differing in the number of interventions.

**Methods:**

We developed a Markov model in which we assumed a healthcare system perspective, 10-year vaccination program and lifetime time horizon. The cohort was the combined African-American and Hispanic 65 year-old birth cohort in the United States in 2009. We evaluated five different vaccination strategies: no vaccination program and four vaccination programs that varied from “low intensity” to “very high intensity” based on the number of interventions deployed in each program, their cumulative cost and their cumulative impact on elderly minority influenza and pneumococcal vaccination rates.

**Results:**

The very high intensity vaccination program ($24,479/quality-adjusted life year; QALY) was preferred at willingness-to-pay-thresholds of $50,000 and $100,000/QALY and prevented 37,178 influenza cases, 342 influenza deaths, 1,158 invasive pneumococcal disease (IPD) cases and 174 IPD deaths over the birth cohort’s lifetime. In one-way sensitivity analyses, the very high intensity program only became cost-prohibitive (>$100,000/QALY) at less likely values for the influenza vaccination rates achieved in year 10 of the high intensity (>73.5%) or very high intensity (<76.8%) vaccination programs.

**Conclusions:**

A practice-based vaccination program designed to eliminate disparities in elderly minority vaccination rates and including four interventions would be cost-effective.

## Background

Influenza and invasive pneumococcal disease (IPD) are vaccine-preventable infectious diseases that together account for >260,000 hospitalizations and >40,000 deaths annually in the U.S. and disproportionately affect the elderly [1-3].

In the elderly, influenza vaccination is recommended annually and the pneumococcal polysaccharide vaccine (PPSV) is recommended at age 65. Both vaccines are effective and widely available [4,5]. Yet, vaccination rates remain far below the *Healthy People 2020* objective of 90%, and there are racial disparities in vaccination rates. In the elderly, 67.7% of Caucasians, 56.1% of African-Americans and 66.8% of Hispanics reported receiving influenza vaccination in 2010 and 63.5% of Caucasians, 46.2% of African-Americans and 39.0% of Hispanics reported having ever received PPSV [6,7]. Given low vaccination rates seen in elderly minority and other populations, many interventions have been studied in an effort to improve vaccination rates [8]. While many studies suggest that single- and multi-component interventions are effective, there are few cost-effectiveness evaluations of these interventions and still fewer cost-effectiveness evaluations of different combinations of interventions [8,9]. This is an important issue for several reasons. First, practices motivated to adopt new strategies to increase elderly minority vaccination rates have a menu of effective interventions from which they may choose, based on Task Force for Community Preventive Services guidelines [8,10]. Second, while it is clear that multi-component interventions are more effective than single-component interventions, it is unclear if they are also more cost-effective [8,9]. Thus, for practices seeking to eliminate disparities in elderly minority vaccination rates, there is little guidance as to which intervention or combination of interventions is likely to be most cost-effective.

Prior exploratory analyses have suggested that vaccination programs to eliminate disparities in elderly minority influenza and pneumococcal vaccination rates are cost-effective when programs cost less than $9-11 per targeted elder per year and result in minority vaccination rates that match Caucasian vaccination rates [11,12]. Here, we extend these exploratory analyses by evaluating the cost-effectiveness of four hypothetical vaccination programs designed to eliminate racial disparities in elderly influenza and pneumococcal vaccination in the outpatient setting and differing primarily in “intensity” (i.e., the number of interventions included in each program).

## Methods

### Model structure

**Figure 1:**
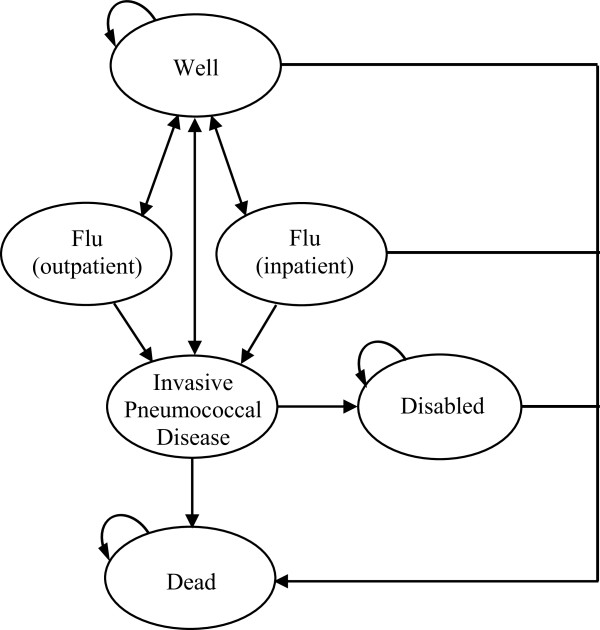
**The Markov state transition diagram for each of five different vaccination program strategies.** During each one year cycle, well patients could acquire influenza, invasive pneumococcal disease or both and subsequently either recover, become disabled or die. All patients ended each one year cycle in one of the following three health states: well, disabled or dead. Patients in the well and disabled states could also die based on all-cause and disability-associated mortality. The only differences between the no program and the four different vaccination program strategies are the cost of the vaccination program and the probability of receiving influenza and pneumococcal polysaccharide vaccination.

To evaluate the cost-effectiveness of different vaccination programs to eliminate disparities in elderly influenza and pneumococcal vaccination rates, we developed a Markov model with a one year cycle length, 10-year vaccination program and lifetime time horizon using TreeAge Pro 2009 software (Figure 1). In a Markov model, a simulated patient cohort iteratively transitions between different health states over time, incurring associated healthcare costs and decrements to quality or quantity of life. We assumed a healthcare system perspective and incorporated direct medical and direct non-medical costs per published guidelines [13]. Our cohort was the combined African-American and Hispanic 65 year-old birth cohort in the United States in 2009, with differences in influenza and pneumococcal vaccination rates modeled based on population-weighted averages.

**Figure 2:**
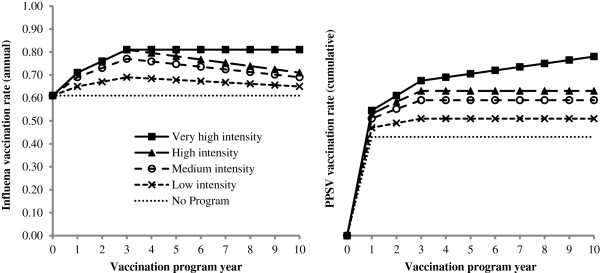
Model assumptions for the five different vaccination program strategies.

We evaluated five different strategies: no vaccination program and four vaccination programs that varied from “low intensity” to “very high intensity” based on the number of interventions deployed in each program, their cumulative cost and their cumulative impact on elderly minority influenza and pneumococcal vaccination rates (Figure 2). Each program incorporated a different number of interventions to increase vaccination rates. Three of these interventions (patient reminders, practice standing orders and practice audit and feedback) were selected for inclusion because they are well-studied, effective and inexpensive [8,14,15]. One intervention (practice vaccination champion) was selected for inclusion as a hypothetical solution to loss of gains in vaccination rates that can occur in later years of an initially high-performing vaccination program due to patient and provider fatigue [16]. A vaccination champion is an individual in the practice who sets vaccination targets, motivates staff and disseminates best practices. We elected to combine interventions in a step-wise fashion because we were primarily interested in evaluating programs that included different numbers of interventions.

In each strategy, we assumed that the cohort entered the model in a “well” state and transitioned to illness states based on probabilities of vaccination, influenza, IPD, hospitalization, disability and death (Figure 1). During each one year cycle, well patients could acquire influenza, invasive pneumococcal disease or both and subsequently either recover, become disabled or die. Patients in the well and disabled states could also die based on all-cause and disability-associated mortality. All patients ended each one year cycle in one of the following three health states: well, disabled or dead. Only patients hospitalized with influenza incurred any risk of influenza-associated mortality or increased risk of IPD. Given IPD’s severity, we assumed that all individuals with IPD were hospitalized. In the absence of available data, we employed a previously described approach and used meningitis rates as a proxy for disability rates for patients hospitalized with invasive pneumococcal diseases and widely varied this parameter in sensitivity analyses [12]. Mortality due to other causes was modeled based on 2009 U.S. life tables for the population-weighted elderly minority cohort [17,18]. The only differences among the five program strategies were the cumulative intervention costs and the probability of receiving vaccines.

### Vaccination program parameters

In the no program strategy, we assumed that 61% received the influenza vaccination each year and 43% received the PPSV in year 1 based on the population-weighted average of elderly African-Americans and Hispanics reporting having received the influenza vaccination in 2010–2011 and having ever received PPSV in 2010, respectively (Figure 2) [6,7,18]. In the no program strategy alone, we assumed that the entire cohort that reported ever receiving PPSV would be vaccinated in year 1 of the model and that there would be no additional PPSV use in subsequent years. This assumption is conservative (i.e. biased in favor of the no program strategy) because it concentrates the benefits of vaccination uptake among younger, healthier patients with more quality-adjusted life years (QALYs) at risk.

In the low intensity program (patient reminders), we assumed that the program cost $2.00 per targeted elder per year, reflecting literature estimates for the costs of autodialed reminders, and caused an 8% absolute increase in influenza and pneumococcal vaccination rates by program year 3, with gradual influenza vaccination rate declines and PPSV rate flattening in program years 3–10 (Figure 2) [8,19]. Between years 3–10, we modeled a 50% loss of the year 1–3 gains in influenza vaccination rates based on author estimates of program fatigue and varied this parameter widely in sensitivity analyses [16]. These vaccination rate trajectories are conservative in that they assume that the 8% increases in year 1 reported in the literature were not achieved until program year 3 and vaccination rate declines occurred in years 3–10 despite continued vaccination program expenditures. We assumed identical vaccination rate trajectories for the medium and high intensity programs, although with greater peak vaccination rate gains by year 3 (Figure 2). The cost per targeted elder per year of the autodialed reminders reflects the sum of time, equipment and supply costs to develop an autodialed vaccination reminder system in a clinic setting [19].

In the medium intensity program (patient reminders and standing orders), we assumed that a standing order intervention added $5.62 in costs per targeted elder per year, reflecting literature estimates for the nursing labor costs associated with screening and identifying patients eligible for vaccination, and caused a 16% absolute increase in vaccination rates by program year 3 [8,20]. This increase in vaccination rates is based on a systematic review of multi-component interventions used to increase vaccination rates that included patient reminders [8]. The cost per targeted elder per year of the standing order intervention reflects the cost of nursing time to screen and identify patients eligible for vaccination and evaluate the patient’s willingness to be vaccinated reported in a prior economic analysis of an inpatient standing order intervention for pneumococcal vaccination [20].

In the high intensity program (patient reminders, standing orders and audit and feedback), we assumed that an audit and feedback intervention added $1.99 in costs per targeted elder per year and caused a 20% absolute increase in vaccination rates by program year 3 [8,14]. Given the absence of published cost data on audit and feedback interventions, we estimated costs based on annual programming costs ($5,060) for the University of Pittsburgh Medical Center electronic medical record system for a network of clinics serving 30,000 patients in which 8.5% of patients in these clinics are minority elders and varied this estimate widely in sensitivity analyses. This approach assumes that audit and feedback intervention costs primarily reflect costs of electronic medical record programming to gather and deliver vaccination progress report cards to physicians. We conservatively allocated costs of the audit and feedback intervention only to minority elders in this clinic network. Because the audit and feedback intervention was added to a program already containing two other component interventions, we conservatively used a lower-bound literature estimate for incremental absolute gains in the vaccination rate (4%) due to audit and feedback interventions [8,14]. This absolute gain is materially lower than median absolute gains in vaccination rates associated with audit and feedback interventions for dichotomous outcomes reported in systematic reviews (16-17%) to reflect concerns regarding the independence of effects of multiple interventions on vaccination rates [8,14]. We then widely varied the incremental absolute gains in the vaccination rate gains associated with the high intensity vaccination program in sensitivity analysis.

In the very high intensity program (patient reminders, standing orders, audit and feedback and vaccination champion), we assumed that a practice vaccination champion would add $8.23 in costs per targeted elder per year based on author estimates that a medical assistant employed by a clinic in the network described above would dedicate one hour per week to leading the vaccination program at an hourly wage cost of $14.51 with an additional 20% in fringe benefits [21]. Again, we allocated costs of the vaccination champion only to minority elders in the clinic network described above and not to all elders. Based on author estimates, we assumed that the practice champion had no added effect on vaccination rates in year 1–3 of the model but prevented declines in influenza vaccination rates and caused an annual 1.5% increase in PPSV rates in years 1–10 of the program. The vaccination rates achieved in all four vaccination programs are reasonable in the context of elderly minority vaccination rates that have been achieved elsewhere for influenza (68-80%) and PPSV (73-77%) [22]. Given uncertainty regarding the incremental cost and effectiveness of a practice vaccination champion, we varied these parameters widely in sensitivity analyses (Table 1).

**Table 1:** Parameter values for base case and sensitivity analyses

		**Parameter range**			
**Description**	**Base**	**Low**	**High**	**Distribution**	**Source**
**Probabilities**					
Influenza and PPSV^a^ vaccination	Figure 2	-50%	+50%	Triangle	[Figure 2]
Vaccination side effects (local reactions)	0.13	0.06	0.20	Beta	[23]
Influenza					
Annual risk	0.10	0.03	0.21	Beta	[24]
Vaccine effectiveness	0.58	0.34	0.74	Beta	[25]
Clinic visit, given influenza	0.62	0.52	0.72	Beta	[26]
Hospitalization, given influenza	0.04	0.01	0.07	Beta	[26]
Increased risk of IPD^b^ given influenza	0.10	0.08	0.13	Beta	[24]
Death given influenza hospitalization	0.23	0.18	0.28	Beta	[1,2]
IPD					
Incidence, disability, mortality	Table 2	-20%	+20%	Triangular	[3], Estimate
Immunocompromised in cohort (%)	Table 2	-20%	+20%	Triangular	[3], Estimate
Vaccine serotype coverage (%)	Table 2	-20%	+20%	Triangular	[3], Estimate
PPSV vaccine effectiveness (yr post-vaccination)				Triangular	[27]
Year 1	0.80	0.60	0.90		
Year 5	0.58	0.31	0.80		
Year 10	0.00	0.00	0.10		
Excess mortality due to disability (per year)	0.1	0.0	1.0	Triangular	[24]
**Costs**					
Vaccination program, per targeted elder per year	Figure 2	-50%	+50%	Triangular	[Figure 2]
Influenza vaccine and administration	$20.97	$13.11	$28.83	Gamma	[28]
PPSV and administration	$33.47	$16.74	$55.79	Gamma	[24]
Vaccine side effect treatment	$0.76	$0.68	$4.01	Gamma	[28]
Influenza and IPD symptomatic treatment	$5.00	$0.00	$10.00	Gamma	[Estimate]
Influenza					
Seeking clinic care	$67.19	$56.62	$77.76	Gamma	[28]
Clinic visit	$158.72	$120.51	$196.92	Gamma	[28]
Hospitalization without death	$5,001	$4,714	$5,406	Gamma	[28]
Hospitalization with death	$10,244	$9,432	$11,173	Gamma	[28]
IPD					
Hospitalization without death	$27,357	$25,224	$30,093	Gamma	[24]
Hospitalization with death	$37,688	$33,919	$41,458	Gamma	[24]
Disability (annual)	$12,683	$10,451	$14,914	Gamma	[29]
**Durations**					
Vaccine side effects (days)	3	1	8	Gamma	[30]
Influenza, outpatient (days)	7	3	10	Gamma	[31]
Influenza, prior to seeking inpatient care (days)	2	1	3	Gamma	[Estimate]
Influenza, inpatient (days)	7	4	10	Gamma	[1]
IPD inpatient (days)	12	9	15	Gamma	[32]
**Utilities**					
One year of healthy life for >65 yr old (QALY)				Uniform	[33,34]
65-70 years	0.76	0.71	0.81		
70-75 years	0.74	0.69	0.79		
75-80 years	0.70	0.65	0.75		
80-85 years	0.63	0.58	0.68		
>85 years	0.51	0.46	0.56		
Vaccine side effects	0.95	0.71	1.00	Uniform	[28]
Influenza, outpatient	0.65	0.49	0.81	Uniform	[28]
Influenza, inpatient	0.50	0.38	0.63	Uniform	[28]
IPD, inpatient	0.20	0.15	0.25	Uniform	[32]
IPD, disabled	0.40	0.20	0.60	Uniform	[35], Estimate

^a^Pneumococcal polysaccharide vaccine.

^b^Invasive pneumococcal disease.

### Vaccine effectiveness

For influenza, we assumed that vaccination reduced influenza risk only in the year of vaccination and that there were no changes in vaccine effectiveness with age (Table 1). For PPSV, we assumed that vaccination effectiveness was a function of baseline effectiveness, declining effectiveness with time, pneumococcal serotype coverage and the percentage immunocompromised (Table 2). Due to the absence of available data, declines in PPSV effectiveness with time were modeled based on expert panel estimates and varied widely in sensitivity analyses [36]. We assumed that PPSV was effective only against IPD and not against non-invasive pneumococcal pneumonia [5].

**Table 2:** Epidemiology of invasive pneumococcal disease (IPD) in the U.S. elderly population, 2007-2008

	**Age cohorts**			
	**65-69**	**70-79**	**≥80**	**Source**
IPD cases per 100,000 per year in the general population (all races)	25.9	33.9	60.1	[ABCs^a^]
African-American population	41.6	54.5	96.4	[Estimate^bc^]
Hispanic population	34.0	44.6	78.9	[Estimate^bd^]
African-American, Hispanic weighted average	38.2	50.0	88.6	[Estimate^be^]
IPD outcomes per 100,000 per year in the general population (all races)				
IPD meningitis	1.6	1.3	1.3	[ABCs]
IPD death	2.9	3.9	11.9	[ABCs]
Pneumococcal polysaccharide vaccine serotype coverage (all races)	74.1%	65.8%	62.9%	[ABCs]
Population immunocompromised (all races)	13.1%	20.2%	23.8%	[ABCs]

^a^ABCs: Active Bacterial Core Surveillance (ABCs) network, 2007–2008.

^b^Estimates of race-level IPD incidence do not include corrections for prior vaccination status explained in the text.

^c^Based on ABCs population level data on incidence of IPD in the African-American population.

^d^Based on relative incidence of IPD in African-American and Hispanic pediatric populations [37].

^e^Based on incidence of IPD in African-American and Hispanic elderly populations and relative population sizes [18].

### Influenza and IPD incidence

We assumed a 10% annual risk of influenza and a 4% hospitalization risk in influenza (Table 1). We modeled age-specific estimates of IPD incidence, disability and mortality based on 2007–2008 data from the Centers for Disease Control and Prevention’s (CDC) Active Bacterial Core Surveillance network (Table 2). As IPD incidence varies by race, we incorporated race-specific IPD estimates. Elderly African-American IPD incidence was estimated for each elderly age cohort by applying the African-American proportion of all IPD cases against the total IPD incidence rates in each age cohort. In the absence of data, we estimated elderly Hispanic IPD incidence based on relative IPD incidence in pediatric African-American and Hispanic populations (1.22:1) and relaxed this assumption in sensitivity analyses [37]. We then calculated a population-weighted average IPD incidence for the cohort based on the relative size of the elderly African-American and Hispanic populations [18]. To account for reductions in IPD incidence rates due to current PPSV use, we adjusted elderly minority IPD incidence rates at the age-cohort level using prior estimates of IPD incidence reductions due to PPSV [38]. This correction provides an estimate of IPD risk if PPSV did not exist. In one-way and probabilistic sensitivity analyses, IPD incidence, IPD disability, IPD mortality, vaccine serotype coverage and percentage immunocompromised were varied across triangular distributions, based on maximum variation reported by the CDC’s ABCs network between 2003 and 2010 (Table 1).

### Costs and effectiveness

We measured costs in 2011 U.S. dollars, inflating prior costs using the U.S. Consumer Price Index. We modeled costs of the following: vaccination interventions, vaccine dose and administration, vaccine side effect treatment, outpatient care, hospitalization and disability (Table 1). Costs were drawn from the medical literature, with hospitalization costs reflecting National Inpatient Survey data [24]. We measured effectiveness in QALYs, assuming that QALYs were a function of the time spent in a given health state multiplied by the utility value of that health state. For patients with influenza requiring only outpatient care, influenza requiring inpatient care and invasive pneumococcal disease requiring inpatient care, we multiplied the time spent in each health state (converting days to years) by the utility value of that health state after adjusting for baseline utility of one year of life for the 65 year old birth cohort (Table 1). Utility values ranged from 0 (death) to 1 (perfect health), were defined by activity limitation and perceived health and were based on data from the 1990 National Health Interview survey [33]. All costs and utilities were discounted at 3% per year per the guidelines of the U.S. Panel on Cost-Effectiveness in Health and Medicine [13].

### Analyses

We performed a cost-effectiveness analysis to determine the incremental cost-effectiveness ratio (ICER) of each vaccination program strategy. Although a $50,000/QALY threshold is commonly used to define cost-effectiveness, a $100,000/QALY threshold is also reasonable [39]. Thus, we identified the programs that were preferred at both willingness-to-pay thresholds. We then performed one-way and probabilistic sensitivity analyses to evaluate the robustness of the model to parameter variation, with results for the probabilistic sensitivity analysis displayed as a net monetary benefit acceptability curve.

## Results

### Base case analysis

The low, medium, high and very high intensity vaccination program strategies – including all illness costs – were progressively more costly and more effective (Table 3). The ICERs of the low, high and very high intensity programs were, respectively, $358, $9,397 and $24,479/QALY. The ICER of the medium intensity program was $11,040/QALY but this strategy was eliminated from consideration because its ICER was higher than that of the more effective high intensity program [13]. The very high intensity program ($24,479/QALY) was preferred at willingness-to-pay-thresholds of both $50,000 and $100,000/QALY. Over the lifetime of the birth cohort, the very high intensity program would be expected to prevent 37,178 influenza cases, 342 influenza deaths, 1,158 IPD cases and 174 IPD deaths (Table 4).

**Table 3:** Base case results

**Vaccination program**	**Cost**	**Incr. cost**	**Effectiveness**	**Incr. eff.**	**ICER**
No program	$698.00		9.3615		
Low intensity	$698.90	$0.90	9.3641	0.0025	$358
Medium intensity	$727.00	$28.10	9.3666	0.0025	Extended^a^
High intensity	$734.80	$36.00	9.3679	0.0038	$9,397
Very high intensity	$776.90	$42.00	9.3696	0.0017	$24,479

^a^Extended dominance: other more effective strategies have a lower ICER; per guidelines this strategy was eliminated [13].

**Table 4:** Estimated public health impact of elderly minority influenza and pneumococcal vaccination programs

	**Influenza**				**Invasive pneumococcal disease**			
		**Cases**		**Deaths**		**Cases**		**Deaths**
**Vaccination program**	**Cases**	**Prevented**	**Deaths**	**Prevented**	**Cases**	**Prevented**	**Deaths**	**Prevented**
No program	457,743		4,211		3,124		471	
Low intensity	445,909	11,834	4,102	109	2,843	281	430	42
Medium intensity	434,072	23,671	3,993	218	2,559	565	388	83
High intensity	428,152	29,591	3,939	272	2,415	709	367	105
Very high intensity	420,565	37,178	3,869	342	1,966	1,158	298	174

Analysis assumed a 65 year old minority birth cohort in the United States, 10 year vaccination program and lifetime time horizon.

### One-way sensitivity analysis

The ICER of the very high intensity program remained < $50,000/QALY across all parameter ranges tested except at less likely values for the PPSV or influenza vaccination rates achieved in the high or very high intensity programs or for the cost of the very high intensity program (Table 5). The ICER of the very high intensity program remained below < $100,000/QALY across all parameter ranges tested except when the peak influenza vaccination rate achieved in the high and very high intensity program was, respectively, >73.5% or <76.8%.

**Table 5:** Results of one-way sensitivity analyses

	**Parameter values**		
**Parameter**	**Base case**	**ICER > $50,000/QALY**	**ICER > $100,000/QALY**
PPSV^a^ vaccination rate, very high intensity program (yr 10)	78.0%	<68.0%	n/a^b^
Cost of very high intensity program (per elder per year)	$17.84	>$23.65	n/a^b^
PPSV vaccination rate, high intensity program (yr 10)	63.0%	>71.7%	n/a^b^
Influenza vaccination rate, very high intensity program (yr 10)	81.0%	<78.2%	<76.8%
Influenza vaccination rate, high intensity program (yr 10)	71.0%	>72.7%	>73.5%

^a^Pneumococcal polysaccharide vaccine.

^b^Maximum variation in these parameters did not cause ICER of preferred program to cross $100,000/QALY threshold.

Results presented only for those parameters, ordered from most to least impact, causing the incremental cost-effectiveness ratio (ICER) of the very high intensity vaccination program to cross $50,000 and $100,000/QALY willingness-to-pay thresholds.

### Probabilistic sensitivity analysis

**Figure 3:**
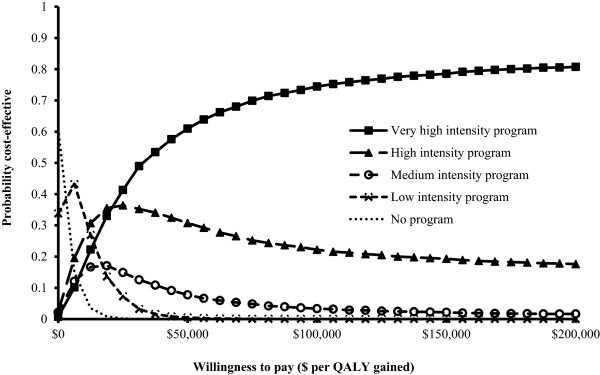
**Probabilistic sensitivity analysis for the five different vaccination program strategies.** Results are displayed in the form of a net monetary benefit acceptability curve.

Results from the probabilistic sensitivity analysis are displayed in Figure 3 in the form of a net monetary benefits acceptability curve showing the probability of the five vaccination program strategies being cost-effective versus the societal willingness-to-pay threshold. Higher intensity vaccination program strategies were preferred at higher willingness-to-pay thresholds. The very high intensity program was most likely to be cost-effective at willingness-to-pay thresholds of greater than $21,000/QALY.

The expected value of perfect information (EVPI), a measure of decision uncertainty, was $24 and $27 per person affected by the decision at thresholds of $50,000 and $100,000 per QALY gained respectively. Assuming a minority elderly population affected by the decision of 8 million over a 10 year time horizon, the population EVPI is $192 to 216 million.^20^ The expected value of partially perfect information, which estimates the contribution of parameters to the EVPI, shows that uncertainties regarding vaccination program costs and effectiveness comprise >90% of the EVPI, due in part to the wide ranges they were assigned. Thus, further research to remove uncertainty regarding disparity program effects on vaccination rates and the costs of such programs could be considered if research costs were less than $167 to 194 million.

## Discussion

Employing conservative assumptions, we found that a very high intensity program deployed to eliminate disparities in influenza and PPSV vaccination rates in elderly minorities in the United States would be cost-effective ($24,479/QALY). Further, in one-way sensitivity analyses, the very high intensity program only became cost-prohibitive (>$100,000/QALY) at less likely values for two parameters.

This study adds an important level of detail to our understanding of vaccination program cost-effectiveness by showing that multi-component vaccination programs designed specifically to eliminate disparities in vaccination rates are likely to be cost-effective [8,19,20,40]. This finding is in accordance with prior work suggesting that programs to eliminate disparities in influenza and pneumococcal vaccination rates would be cost-effective if they eliminate disparities in elderly minority vaccination rates and cost < $9-11 per targeted elder per year [11,12]. Here, we found that the ICER of the very high intensity program ($24,479) was lower than the ICERs of minority vaccination programs described previously ($45,000-$48,000/QALY) [11,12]. These differences are likely due to the very high intensity program resulting in more vaccinations than previous programs (influenza and PPSV together v. either vaccination alone) [11,12].

This study has several strengths. First, we incorporated conservative assumptions regarding the costs and effectiveness of the four vaccination programs, assuming that costs were assigned to each targeted minority elder per year regardless of prior PPSV vaccination status, that single year gains in vaccination rates reported in the literature were only realized by the third year of the program, and that gains in vaccination rate either stagnated (PPSV) or were lost (influenza) in years 3–10 for all but the very high intensity vaccination program. Second, we made no assumptions regarding the potential for this elderly minority vaccination program to also increase vaccination rates in the elderly Caucasian population seen in the same practices. Third, the results were robust to wide variation in parameter values, with the very high intensity program not becoming cost-prohibitive (>$100,000/QALY) except when less likely values were tested in sensitivity analyses for the influenza vaccination rate achieved in year 10 of either the high intensity or the very high intensity vaccination program.

This study also has several limitations. First, the study evaluated the cost-effectiveness of four practice-based vaccination programs designed to eliminate disparities in influenza and pneumococcal vaccination rates. In theory, this modeling choice limits the generalizability of our findings to those practices or practice networks that serve a large population of minority elders, have disparities in vaccination rates and have not adopted the interventions described above. In practice, however, this study is likely generalizable to any practice with elderly influenza and pneumococcal vaccination rates that are less than or equal to the baseline elderly minority vaccination rates modeled in this study. Second, in the absence of better data, we modeled declines in PPSV vaccine effectiveness based on expert panel estimates and costs of audit and feedback and practice vaccination champion interventions based on author estimates. When varied widely in sensitivity analyses, however, these parameters did not have a large impact on the ICER of the preferred vaccination program. Third, our modeling approach does not capture vaccination effects in decreasing disease transmission to susceptible unvaccinated persons, as would a dynamic transmission model. However, since our targeted group, the elderly, is not the core group for transmission in these illnesses (as children are), these transmission effects would likely be minimal.

## Conclusions

This analysis suggests that a very high intensity vaccination program that included four interventions to eliminate disparities in elderly minority influenza and PPSV vaccination rates would be cost-effective at both $50,000 and $100,000/QALY willingness-to-pay thresholds. A vaccination program including these four interventions should be considered for adoption in community-based practices seeking to address disparities in elderly minority vaccination rates. Further research on the costs and effectiveness of such programs may be warranted and could be undertaken in the form of a large pragmatic clinical trial that would likely be economically reasonable to conduct given the value of the information garnered from such a trial.

## Abbreviations

CDC: Centers for disease control and prevention; ICER: Incremental cost-effectiveness ratio; IPD: Invasive pneumococcal diseases; PPSV: Pneumococcal polysaccharide vaccine; QALY: Quality-adjusted life year.

## Competing interests

CIM has no conflicts to report. RKZ has received grant support from Merck, Sanofi and MedImmune and received consulting fees from MedImmune. MPN has received grant support from Merck and MedImmune and received consulting fees from MedImmune. KJS has received grant support from the St. Margaret’s Foundation via grant support from Merck to study the cost-effectiveness of the pneumococcal polysaccharide vaccine.

## Authors’ contributions

CIM carried out the cost-effectiveness analysis and drafted the manuscript. MPN, RKZ and KJS conceived of the study, participated in its design and coordination and helped draft the manuscript. All authors read and approved the final manuscript.

## Pre-publication history

The pre-publication history for this paper can be accessed here:

http://www.biomedcentral.com/1471-2458/14/718/prepub
